# The last week of life of nursing home residents with advanced dementia: a retrospective study

**DOI:** 10.1186/s12904-019-0510-x

**Published:** 2019-12-27

**Authors:** Franco Toscani, Silvia Finetti, Fabrizio Giunco, Ines Basso, Debora Rosa, Francesca Pettenati, Alessandro Bussotti, Daniele Villani, Simona Gentile, Lorenzo Boncinelli, Massimo Monti, Sandro Spinsanti, Massimo Piazza, Lorena Charrier, Paola Di Giulio

**Affiliations:** 1 Lino Maestroni Foundation, Palliative Medicine Research Institute, via Palestro 1, 26100 Cremona, Italy; 2 Department of Health and Social Services Polo Lombardia 2, Don Carlo Gnocchi Foundation ONLUS, Via Palazzolo, 21, 20149 Milan, Italy; 3 Intensive Care Unit, SS Antonio e Biagio e Cesare Arrigo Hospital, Via Venezia, 16, 15121 Alessandria, Italy; 4 University of Milan, section of Don Carlo Gnocchi Foundation, Via A. Capecelatro, 66, 20148 Milan, Italy; 5 Agenzia Continuità Ospedale Territorio, Azienda Ospedaliero- Universitaria Careggi, Largo Brambilla 3, 50134 Florence, Italy; 6 Neuro-Rehabilitation and Alzheimer Disease Evaluation Unit, Figlie di San Camillo Hospital, Via F. Filzi , 56, 26100 Cremona, Italy; 7 Rehabilitation and Alzheimer Disease Evaluation Unit, Ancelle della Carità Hospital, Via G. Aselli, 14 Cremona, Italy; 8 Intensive Care Unit Geriatric, AOU Careggi-Largo Brambilla,3, 50134 Florence, Italy; 9 Geriatric Institute Pio Albergo Trivulzio, via Trivulzio, 15, 20146 Milan, Italy; 10 Istituto Giano, Via Stazzo Quadro 7, 00060 Riano (Rm), Milan, Italy; 11 Italian Foundation of Leniterapia (FILE), Via San Niccolò, 1, 50125 Florence, Italy; 12 Department of Public Health and Pediatrics, University of Turin, Via Santena 5 bis, 10126 Turin, Italy; 13 SUPSI, Manno, Switzerland

**Keywords:** Nursing homes, Dementia, Palliative care

## Abstract

**Background:**

Barriers to palliative care still exist in long-term care settings for older people, which can mean that people with advanced dementia may not receive of adequate palliative care in the last days of their life; instead, they may be exposed to aggressive and/or inappropriate treatments. The aim of this multicentre study was to assess the clinical interventions and care at end of life in a cohort of nursing home (NH) residents with advanced dementia in a large Italian region.

**Methods:**

This retrospective study included a convenience sample of 29 NHs in the Lombardy Region. Data were collected from the clinical records of 482 residents with advanced dementia, who had resided in the NH for at least 6 months before death, mainly focusing on the 7 days before death.

**Results:**

Most residents (97.1%) died in the NH. In the 7 days before death, 20% were fed and hydrated by mouth, and 13.4% were tube fed. A median of five, often inappropriate, drugs were prescribed. Fifty-seven percent of residents had an acknowledgement of worsening condition recorded in their clinical records, a median of 4 days before death.

**Conclusions:**

Full implementation of palliative care was not achieved in our study, possibly due to insufficient acknowledgement of the appropriateness of some drugs and interventions, and health professionals’ lack of implementation of palliative interventions. Future studies should focus on how to improve care for NH residents.

## Background

Dementia is an incurable condition that causes a progressive decline in health, characterized by comorbidities, increased severity of physical and cognitive disabilities, acute conditions typical of frailty, the worsening of chronic comorbidities, and dementia-related events such as recurrence of infections and eating problems, all of which require specific palliative care strategies [1]. People with dementia can survive for years [2] and are usually cared for in residential facilities, in particular in nursing homes (NHs), which provide many or all of the long-term care services they need until their death [3]. Thus, for most of their residents, NHs should be prepared and able to provide palliative care.

However, barriers to palliative care still exist in long-term care settings for older people [4], which can mean that people with advanced dementia may not receive adequate palliative care in the last days of their life; instead they may be exposed to aggressive and/or inappropriate treatments [5–7]. Lack of communication between health care professionals and family carers is a barrier to the implementation of palliative care as family carers may be reluctant to accept that their relative is in the dying phase [1]. However, the family’s understanding of the disease progression and of the dying phase does not always guarantee the implementation of palliative care [8]. Other barriers are high staff turnover (especially nurses) [9]; limited number of nurses [10]; and lack of competence in palliative care [11, 12]. Important differences in palliative care delivery do exist across countries [13], and also among institutions within the same country [14]. Italian NHs differ widely in the number of beds they have, the services they offer, and their quality of care, but very few provide palliative care consultations [6, 15].

## Methods

### Aim

The aim of this multicentre study was to assess the clinical interventions and care at end of life in a cohort of NH residents with advanced dementia in a large Italian region.

### Study setting

We used data from the Valuazione dell’Efficacia della Leniterapia nell’Alzheimer e Demenze (VELA Project) [16], which was conducted in collaboration with the Fondazione Italiana Leniterapia of Florence and the Lino Maestroni Palliative Medicine Research Foundation of Cremona. The aim of the VELA Project was to compare end-of-life care procedures provided to NH residents with advanced dementia in the Lombardy Region, and in the surrounding areas of Florence in the Tuscany Region, before and after a short educational intervention to improve palliative care. Due to regional differences in the organization of NHs and in the services provided to NH residents with dementia, here we present only data from the Lombardy Region.

In the Lombardy Region, accredited NHs have their own medical and nursing staff, with a regulatory requirement ratio of ≥901 min/week of care per resident [17]; these NHs may be defined as “skilled NHs”. With few exceptions, there is at least one nurse with a bachelor’s degree available per shift. NHs in the region may also rely on consultants (geriatricians, neurologists, physiatrists, etc.) and may employ psychologists, occupational therapists, physiotherapists, or other specialists. Out of a network of 34 NHs in the Lombardy Region that participated in a previous study [15], 29 agreed to participate in the present study (number of beds per NH ranged from 40 to 714). To be included in the analysis, NH residents had to have a Functional Assessment Staging Tool (FAST) stage ≥7c (double incontinence; loss of all intelligible vocabulary; non-ambulatory) [18] and have resided in the NH for at least 6 months before death.

The study was approved by the ethics committee of Don Carlo Gnocchi Foundation of Milano on February 20, 2013; it started on April 1, 2013 and concluded on January 31, 2015. In this paper, we describe the 60 days before death, with a special focus on the last 7 days in the entire cohort (pre and post educational intervention) of NH residents. Due to the limited impact of the educational intervention [16], the two cohorts were merged.

### Data collection

Trained monitors collected data from clinical records, nursing records, and drug data sheets on up to 20 residents in each of the 29 participating NHs. This information included demographic characteristics, major comorbidities, and cause of death, as well as presence of the following: a comprehensive evaluation of the severity of clinical conditions, advance care planning, a legal representative (guardian), do not resuscitate (DNR) and do not hospitalize (DNH) orders, NH resident’s wishes about treatment and funeral dispositions, and any other advance directive (AD). Specific information was also collected on nutrition and hydration in the 60 and 7 days before death, as well as on dialysis, symptoms, tests and interventions administered such as endotracheal suctioning, hospitalizations and admissions to the emergency department, assessment of pain and discomfort, drugs prescribed (classified according to the Anatomical Therapeutic Chemical code) [19], and palliative sedation in the 7 days before death. Nutrition and hydration were classified by a panel of experts (including experts in palliative care, geriatrics, nursing, psychology, family medicine, and bioethics) as palliative-oriented nutrition if nutrition and/or hydration were given by mouth only, if nutrition by mouth was accompanied by comfort hydration (i.e. the administration of < 1000 ml of fluids/day by subcutaneous hydration), if only subcutaneous hydration was used, or if no nutrition or hydration was provided at all [16]. Comfort hydration was seen as a compromise between the advisability to reduce water intake to improve comfort and reduce symptoms, and family members’ expectations and beliefs about hydration [20]. Nutrition and hydration were classified as non-palliative-oriented nutrition if given by parenteral route, via nasogastric tube, or via percutaneous endoscopic gastrostomy at any point during the 7 days before death [16]. Drugs prescribed in the 7 days before death were collected from drug sheets. NH residents with missing drug sheets and those who were admitted to hospital or the emergency department in the 7 days before death were excluded from the analyses on drugs prescribed.

### Statistical analyses

For categorical variables, data are shown as absolute and relative (%) frequencies with 95% confidence intervals (CIs). Mean and standard deviation or median and interquartile range (IQR), as appropriate, were calculated for continuous variables. All analyses were performed with Stata 14 (StataCorp., College Station, TX, USA).

## Results

A cohort of 482 NH residents was recruited, 26.8% of whom had Alzheimer-type dementia. Overall, half of the residents had between five and eight comorbidities (median 6), and 25% had more than eight comorbidities (Table 1).

**Table 1:** Main characteristics of the 482 nursing home (NH) residents with advanced dementia (Functional Assessment Staging Tool stage ≥7c)

	n (*n* = 482)	%	95% CI^b^ (%)
Sex Female	365	75.7	71.9;79.5
Age at NH admission, years, median (IQR^b^): 84.1 (79–88.7)			
Diagnosis of dementia			
Alzheimer	129	26.8	22.8;30.7
Not defined	175	36.3	32.0;40.6
Vascular	138	28.6	24.6;32.7
Mixed	33	6.8	4.6;9.1
Lewy body	7	1.5	0.4;2.5
Comorbidities			
Genitourinary	435	90.2	87.6;92.9
Musculoskeletal	409	84.9	81.6;88.0
Gastrointestinal tract	135	28.0	24.0;32.0
Peripheral and central nervous system	314	65.1	60.9;69.4
Hypertension	238	49.4	44.9;53.8
Cardiovascular	237	49.2	44.7;53.6
Head and neck	207	42.9	38.5;47.4
Vascular	195	40.5	36.1;44.8
Respiratory	100	20.7	17.2;24.4
Endocrine-metabolic	135	28.0	24.0;32.0
Kidney	55	11.4	8.6;14.2
Liver	30	6.2	4.1;8.4
Others^a^	18	3.7	2.0;5.4

^a^Others: other cancers [15]; anemia [2]; pressure sores [1]

^b^*CI* Confidence interval, *IQR* Interquartile range

Almost all residents (468, 97.1%) died in the NHs (median age at death: 89 years, IQR 83.6–93.1), 69 (14.7%) of them had a family member present during the last hours of life (this information was available for 358 residents). Cause of death was available for 374 (77.6%) NH residents, 96 (25.7%) of whom had dementia reported as the cause of death. Only one NH resident had a self-written AD; in 19 (3.9%) cases, a family member reported the NH resident’s wishes; and 60 (12.4%) residents had guardian. In six (1.2%) cases, the family reported the resident’s preference for cremation.

In the 60 days before death, 378 (78.4%) NH residents were fed only by mouth; 43 (8.9%) were fed by mouth and intravenous or subcutaneous integration; and 43 (8.9%) were tube fed; data where missing for the remaining 18 residents. During the 60 days before death, a new feeding tube was placed in 26 NH residents (of the 63 with a feeding tube in the 7 days before death, 37 already had it at 60 days before death).

### 7 days before death

A substantial worsening of clinical conditions (sometimes defined as “terminal conditions”) was recorded in the clinical records a median of 4 days (IQR 2–11) before death for 275 (57.1%) NH residents, and the notification of impending death was recorded a median of 1 day before death (IQR 0–3) for 150 (31.1%) residents. Advance care planning was drawn up for only 21 NH residents (4.4%) (median 15 days before death; IQR 5–41). Two (0.4%) NH residents had a DNR, seven had a DNH (1.5%), and one NH resident had both.

Two hundred and nine residents’ clinical records included a registration of discussions with families on the worsening of residents’ conditions, which took place a median of 3 (IQR 1–7) days before death. Eighty-six of these records also reported a discussion with the family of decisions to be made, which took place a median of 6 (IQR 2–20) days before death.

After the exclusion of 13 NH residents with missing information on nutrition and hydration, we observed palliative-oriented nutrition in 130 (27.7%) residents (Table 2).

**Table 2:** Nutrition and hydration in the 7 days before death

	n (*n* = 469)^b^	%	95% CI^a^ (%)
IV^a^ hydration (alone or supplement)	227	48.4	43.9;52.9
Nutrition/hydration by mouth only	94	20.0	16.4;23.7
SFA^a^ only (or SFA+ mouth)	70	14.9	11.7;18.1
Tube feeding	63	13.4	10.3;16.5
Parenteral Nutrition	15	3.2	1.6;4.8
Comfort hydration (IV and SFA)	99/455	21.8	18.0;25.5

^a^*CI* Confidence interval, *IV* Intravenous, *SFA* Subcutaneous fluids administration

^b^13 residents with missing information on nutrition and hydration

Overall, 101 NH residents (21%) received at least one invasive treatment or intervention in the 7 days before death (Table 3).

**Table 3:** Invasive treatments/interventions in the 7 days before death

	n (*n* = 482)	%	95% CI^b^ (%)
Oral/tracheal suctioning	101	21.0	17.3;24.6
Blood collection	73	15.1	11.9;18.3
Peripheral vein cannulation (one or more attempts)	53	11.0	8.2;13.8
Insertion/repositioning of urinary catheter	30	6.2	4.1;8.4
Insertion/repositioning of a nasogastric tube (2 PEGs^b^)	11	2.3	0.9;3.6
Insertion of a central venous catheter	3	0.6	0.0;1.3
Other invasive treatments^a^	6	1.2	0.2;2.2

^a^Other treatments (residents could be exposed to more than one treatment): Enema [2]; Glycaemia measurement [2]; Hemogasanalysis [1]; Flu vaccine [1]

^b^*CI* Confidence interval, *PEG* Percutaneous endoscopic gastrostomy

In the 7 days before death, nine residents were sent to the emergency department and then discharged, while 14 were admitted to hospital (2.9%). Pain and/or discomfort were assessed for 192 (39.8%) residents. In 13 (2.7%) cases, palliative pharmacological sedation was provided; 70 residents (14.5%) underwent resuscitation attempts, 62 of which were performed by NH staff: five as cardio-pulmonary resuscitation, and 66 with life-saving drugs.

Data on drugs prescribed were available for 316 (65.6%) NH residents; a median of five (IQR 3–7) drugs were prescribed for these residents in the 7 days before death, and 22.2% NH residents had two or fewer drugs prescribed (Table 4).

**Table 4:** Prescriptions in the 7 days before death classified according to the Anatomical Therapeutic Chemical (ATC) code

	ATC code	n (*n* = 316)	%	95% CI^b^ (%)
Anticoagulants - Antiplatelets	B01A	166	52.5	47.0;58.0
Drugs for acid related disorders	A02	153	48.4	42.9;53.9
Cardiovascular System	C			
Cardiac therapy	C01A, C01D	130	41.1	35.7;46.6
Beta blocking agents	C07	31	9.8	6.5;13.1
Anti-arrhythmic class I-III	C01B	8	2.5	0.8;4.2
Antibiotics (antibacterial/antimycotics)	J01;J02	107	33.9	28.6;39.1
Diuretics	C03	110	34.8	29.5;40.1
Opioids	N02A	108	34.2	28.9;39.4
Laxatives	A06	96	30.4	25.3;35.4
Vitamins and mineral supplements	A11; A12	88	27.8	22.9;32.8
Antipsychotics	N05A	79	25.0	20.2;29.8
Benzodiazepine derivates	N05CD	70	22.2	17.6;26.7
Steroids	H02AB	58	18.4	14.1;22.6
Drugs for the respiratory system	R	37	11.7	8.2;15.2
Antiepileptics	N03	30	9.5	6.3;12.7
Insulin and other glucose lowering	A10	27	8.5	5.5;11.6
Acetaminophen	N02B	24	7.6	4.7;10.5
Antidepressants	N06	24	7.6	4.7;10.5
Anti-parkinson	N04	23	7.3	4.4;10.1
Antiemetics	A04	19	6.0	3.4;8.6
Hyoscine Butylbromide	N07	14	4.4	2.2;6.7
Thyroid drugs	H03	17	5.4	2.9;7.9
Others^a^		29	9.2	6.0;12.4

^a^Others: Allopurinol (M04 11), Drugs for benign prostatic hypertrophy (*Tamlusosin, Finasteride* (G04 AC, 10), Antidiarrhoics (A07, 5), Ursodesooxyicholic acid (A05AA02 4), Drugs for treatment of hyperkaliemia and hyperphosphatemia (V03AE, 3), Hormone antagonists (L02, 2), Baclofen (M03BX, 2); only 1: Epoietine (B03); Rociverine (A03AA)

^b^*CI* Confidence interval

## Discussion

This study presents data on a large cohort of NH residents with advanced dementia who died in a NH located in a large region of northern Italy. In contrast to previous studies, in which one-third of NH residents were hospitalized in the last month of life and the rate of death in hospital was almost 66% [21, 22], the vast majority of our residents died in the NHs: only 4.7% of them were admitted to hospital or sent to the emergency department. This low figure may be considered positive, as hospitalization can be aggressive and of limited clinical benefit for people with advanced dementia [23]. The availability of a full-time physician, and the fact that all NHs that participated in the present analysis are skilled NHs, may account for this result.

In general, although the care received by NH residents with advanced dementia in the 7 days before death showed room for improvement, it also showed a preference for non-aggressive treatment. Sixty days before death, the vast majority of NH residents (78.4%) were fed only by mouth, but, as expected, that number decreased to nearly one in five (20%) in the 7 days before death. Overall, one-third of NH residents received palliative-oriented nutrition in the 7 days before death. It is broadly acknowledged that tube feeding in people with advanced dementia is ineffective and even harmful, and that possible symptoms of dehydration can be effectively treated with small amounts of fluids (by mouth or by subcutaneous fluids administration) with good oral care [24]. Nevertheless, relatives often ask for substantial nutrition and hydration [25]. The limited use of subcutaneous fluids administration in our study could be ascribed to a lack of knowledge of this technique on the part of NH staff [26].

The figures we report related to feeding tubes, namely the number of patients who died with a feeding tube in place, are higher than those reported in a Dutch [27] and a US study, which showed a decrease in tube feeding over 15 years (from 11.7% in 2000 to 5.7% in 2014) [28]. Nevertheless, our results reflect a substantial positive trend when compared with the findings of a previous study (tube feeding 21.0% vs tube feeding and parenteral nutrition 16.6%; intravenous hydration 66% vs 48.4%) carried out in 2005 in a smaller sample of NHs in the same region [5]. The same trend toward a less aggressive approach was shown by the decreased use of intravenous fluid administration (from 67% to nearly 40%) [6]. Further positive findings from our study concern the use of drugs, with the increased use of opioids (from 4.9% at baseline [29] to 34.2% in the 7 days before death) and acetaminophen (from 4.5% [29] to 7.6%); the low rate of blood samples taken (less than 2 patients out of 10); and the low rate of other invasive treatments/interventions.

Some aspects still require closer scrutiny and improvement, in particular clinical factors. For example, the prescribing of inappropriate drugs [30, 31], such as anticoagulants/antiplatelets and anti-arrhythmics classes I-III, continued in our study. Moreover, diuretics, beta-blocking agents, antipsychotics, and antibiotics, even if considered “sometimes appropriate”, seemed to be overly prescribed in the last 7 days before death (hyoscine, steroids, and antacids may have been prescribed as symptomatics). There was also an overuse of procedures like intravenous catheter placement and an underuse of subcutaneous fluids administration.

The acknowledgement of the worsening of resident’s conditions was reported in the clinical records of 57% of NH residents a few days before death (median 4), but only 21 of these residents had a care plan in place. Lastly, considering the residents’ advanced stage of dementia, some questionable resuscitation attempts were carried out (14.5%, mostly with drugs). The trajectories of decline in persons with dementia are uncertain [32]; therefore it is not easy to assess when a resident is approaching death. If the proximity of death is only acknowledged when a resident’s health condition rapidly declines [33], fewer opportunities are left to provide palliative care and hospice referral [34]. The difficulty of defining terminality, and of reliably estimating survival in people with advanced dementia requires structured investments to produce effective tools to identify and evaluate these factors [35]. These findings also show the need to improve the knowledge of NH staff, including physicians, on these issues [16]. This need is reflected in the urgency placed on national regulatory bodies and international scientific boards to produce up-to-date, widely accepted guidelines on the appropriate pharmacological approach to take in patients with dementia who are at the end of their life [7, 30].

Critical decisions also require closer scrutiny and improvement. One of the most disappointing findings was the extremely low presence of ADs, whether they were self-written or communicated by relatives, and the scarcity of guardians and/or surrogates. In Italy, the legal representative must be appointed by a magistrate, generally at the request of the health care provider (e.g., general practitioner and/or the NH doctor), and this procedure can take some time. This may explain the scarcity of DNR and/or DNH orders in our study (overall 8 NH residents) and even the use of palliative pharmacological sedation. DNR and DNH orders are far more common in other European countries: 2.4% in our study, compared to 21.0% in Holland [36] and 60% in the US [37].

Family involvement in decisions was documented in only 86 clinical records, and a recent study reported that most decisions are first taken by the physicians and only later communicated to relatives [15]. The low prevalence of advance care planning suggests a tendency to avoid addressing the issue with family [38], and efforts should be made to improve this. Advance care planning implies the involvement of family, in what have been called “expectation conversations” [39]. Only 3.4% of NH residents were involved in conversations on desired end-of-life care in the van der Voot et al. study [36] (although more than 60% were cognitively competent at admission), and even if NH staff say they are available to speak with NH residents’ relatives about death and dying when they are “terminal” [38], systematic, periodic conversations with families are a crucial instrument to reducing NH staff’s uncertainty in clinical decision-making and to improve the family’s perceptions of quality of care in NHs [39, 40].

Critical decisions may affect the time of death, the place of death [33], and the way a person will live her/his last days. If a patient is incapacitated and there is no surrogate and/or ADs, the ultimate responsibility for treatment decisions falls on the physician, whose decisions may be affected by several factors, such as the context (setting and culture) [41], personal and societal values and constraints, and medical training that is overly focused on curing [15, 42]. A framework that shares this planning with the family may reduce the temptation to revert to the use of defensive medicine (i.e., practices undertaken primarily to avoid liability rather than to benefit the patient), which may be partially responsible for resuscitation attempts, placement of feeding tubes [25], the drawing of blood samples, the avoidance of writing DNR/DNH orders, etc. This behavior implies that, in spite of the spread and seeming acceptance of the principles and methods of palliative care, at least in NHs, prejudices and fears persist.

Our results are limited by the retrospective nature of the study and by data retrieval from clinical records; some aspects such as relatives’ involvement in decisions may have been under-reported. Although this is a multicentre study, each NH contributed the same number of cases, thus limiting the possible over-representation of residents from larger NHs. Data were collected by expert, trained researchers, which may have limited interpretation problems.

## Conclusions

Notwithstanding some clear improvements in the quality of palliative care, which reflects a positive trend toward a less aggressive approach to treatment among NH residents with advanced dementia in their last days of life, there are still some barriers to a full implementation of palliative care. In particular, insufficient acknowledgement of the inappropriateness of some drugs and interventions, and reluctance to implement end-of-life palliative care interventions. In order to provide quality palliative care to NH residents with advanced dementia, up-to-date, widely accepted guidelines on the pharmacological approach to take in patients with advanced dementia are urgently needed. At the same time, changes in the cultural approach to death and dying are necessary in NH staff and the general population if clinically correct care strategies are to be outlined that can be agreed upon by NH residents and their families.

## Data Availability

The datasets used and/or analyzed during the current study are available from the corresponding author on reasonable request.

## Abbreviations

AD: Advance directive
ATC: Anatomical Therapeutic Chemical
CI: Confidence interval
DNH: Do not hospitalize
DNR: Do not resuscitate
FAST: Functional Assessment Staging Tool
IQR: Interquartile range
IV: Intravenous
NH: Nursing home
PEG: Percutaneous endoscopic gastrostomy
SFA: Subcutaneous fluids administration
VELA: Valutazione dell’Efficacia della Leniterapia nell’Alzheimer e Demenze
